# Effect of near-infrared spectroscopy on postoperative delirium in cardiac surgery with cardiopulmonary bypass: a systematic review and meta-analysis

**DOI:** 10.3389/fcvm.2024.1404210

**Published:** 2024-06-18

**Authors:** Qian Sun, Weiguo Wu

**Affiliations:** Department of Anesthesiology, Qilu Hospital (Qingdao), Cheeloo College of Medicine, Shandong University, Qingdao, China

**Keywords:** postoperative delirium, near-infrared spectroscopy, cardiopulmonary bypass, cardiac surgery, cerebral oxygen saturation, meta-analysis

## Abstract

**Background:**

Postoperative delirium (POD) is a common anesthetic side effect in cardiac surgery. However, the role of oxygen saturation monitoring in reducing postoperative delirium has been controversial. Therefore, this meta-analysis aimed to analyze whether NIRS monitoring during cardiac surgery under cardiopulmonary bypass could reduce the incidence of postoperative delirium.

**Methods:**

PubMed, Web of Science, Cochrane Library, Embase and China National Knowledge Infrastructure (CNKI) databases were systematically searched using the related keywords for randomized-controlled trials (RCTs) published from their inception to March 16, 2024. This review was conducted by the Preferred Reporting Project and Meta-Analysis Statement (PRISMA) guidelines for systematic review. The primary outcome was postoperative delirium, and the second outcomes included the length of ICU stay, the incidence of kidney-related adverse outcomes, and the incidence of cardiac-related adverse outcomes.

**Results:**

The incidence of postoperative delirium could be reduced under the guidance of near-infrared spectroscopy monitoring (OR, 0.657; 95% CI, 0.447–0.965; *P* = 0.032; I^2^ = 0%). However, there were no significant differences in the length of ICU stay (SMD, 0.005 days; 95% CI, −0.135–0.146; *P* = 0.940; I^2^ = 39.3%), the incidence of kidney-related adverse outcomes (OR, 0.761; 95% CI, 0.386–1.500; *P* = 0.430; I^2^ = 0%), and the incidence of the cardiac-related adverse outcomes (OR, 1.165; 95% CI, 0.556–2.442; *P* = 0.686; I^2^ = 0%) between the two groups.

**Conclusion:**

Near-infrared spectroscopy monitoring in cardiac surgery with cardiopulmonary bypass helps reduce postoperative delirium in patients.

**Systematic Review Registration:**

PROSPERO, identifier, CRD42023482675

## Introduction

Cardiopulmonary bypass (CPB) has been widely used in cardiac surgery, and its related complications have received increasing attention (1, 2). Postoperative delirium (POD) is a common clinical complication, with an incidence of up to 3.1%–52% (3–6), which may lead to a longer hospital stay, increased social burden, and reduced recovery and quality of life. POD is associated with several factors, including age, type of surgery, preoperative cognitive level of the patient, and other postoperative-related factors (7–9). During cardiac surgery with cardiopulmonary bypass, the detached atherosclerotic plaque and air trapping in the CPB circuit may form emboli, leading to cerebral embolism and increasing the risk of postoperative delirium (10). Inflammation and ischemia-reperfusion injury also play an important role in the pathogenesis of POD (11). In addition, hypothermia, blood pressure fluctuation and internal environment disorder during CPB may affect cerebral perfusion, leading to cerebral ischemia and hypoxia, which may lead to the occurrence of POD (12). Oxygen saturation is one of the most critical factors in POD (13, 14), the changes in cerebral oxygen saturation also indirectly reflect the changes in cerebral perfusion. Studies have shown that 50%–70% of patients undergoing cardiac surgery experience one or more decreases in rSO2 during CPB (15). Near-infrared spectroscopy (NIRS) is widely used to monitor rSO2 in cardiac surgery. As a monitoring device that indirectly reflects the changes between brain metabolism and oxygen supply and demand of patients during surgery, NIRS has the potential to noninvasively assess the balance of oxygen supply and demand of frontal brain tissue and provide real-time local oxygen saturation during cardiopulmonary bypass (16–18).

We conducted this systematic review and meta-analysis to compare and summarize whether the use of cerebral oxygen saturation monitoring during cardiac surgery with cardiopulmonary bypass reduces the incidence of postoperative delirium.

## Methods

This review was conducted by the Preferred Reporting Project and Meta-Analysis Statement (PRISMA) (19) guidelines for systematic review. This systematic review and meta-analysis were included in PROSPERO (registration number: CRD42023482675).

### Search strategy

We systematically searched PubMed, Web of Science, Cochrane Library, Embase and China National Knowledge Infrastructure (CNKI) databases for English articles published from inception to March 16, 2024. The related search terms were as follows: ((((([“Delirium"(Mesh)] OR (((((((Subacute Delirium) OR (Delirium, Subacute)) OR (Deliriums, Subacute)) OR (Subacute Deliriums)) OR (Delirium of Mixed Origin)) OR (Mixed Origin Delirium)) OR (Mixed Origin Deliriums))) AND ([“Cardiac Surgical Procedures"(Mesh)] OR ((((((((((((cardiac surgery) OR (Procedure, Cardiac Surgical)) OR (Procedures, Cardiac Surgical)) OR (Surgical Procedure, Cardiac)) OR (Surgical Procedures, Cardiac)) OR (Surgical Procedures, Heart)) OR (Cardiac Surgical Procedure)) OR (Heart Surgical Procedures)) OR (Procedure, Heart Surgical)) OR (Procedures, Heart Surgical)) OR (Surgical Procedure, Heart)) OR (Heart Surgical Procedure)))) OR ([“Cardiopulmonary Bypass"(Mesh)] OR (((((((Heart-Lung Bypass) OR (Bypass, Heart-Lung)) OR (Bypasses, Heart-Lung)) OR (Heart Lung Bypass)) OR (Heart-Lung Bypasses)) OR (Bypass, Cardiopulmonary)) OR (Bypasses, Cardiopulmonary)))) AND ([“Spectroscopy, Near-Infrared"(Mesh)] OR (((((((((((((Near-Infrared Spectroscopies) OR (Near-Infrared Spectroscopiesoscopy)) OR (Spectroscopies, Near-Infrared)) OR (Spectroscopy, Near Infrared)) OR (NIR Spectroscopy)) OR (NIR Spectroscopies)) OR (Spectroscopies, NIR)) OR (Spectroscopy, NIR)) OR (Spectrometry, Near-Infrared)) OR (Near-Infrared Spectrometries)) OR (Near-Infrared Spectrometry)) OR (Spectrometries, Near-Infrared)) OR (Spectrometry, Near Infrared)))) OR (Cerebral Oxygen Saturation)).

All citations were downloaded and imported into EndNote for management (20). Duplicates were first eliminated, titles and abstracts were reviewed to exclude studies that did not meet the inclusion criteria, and the remaining articles were analyzed and further screened according to the inclusion and exclusion criteria.

### Selection criteria

The inclusion criteria were developed based on the Population, Intervention, Comparison, Outcomes, and Study framework: (1) cardiac surgery patients undergoing CPB; (2) comparator: Cerebral oxygen saturation monitoring group vs. the control group; (3) POD is included in the ending; and (4) the study was randomized controlled trial (RCT). The exclusion criteria were as follows: (1) no original or incomplete data; (2) non-adult cardiac surgery; (3) emergency surgery, aortic dissection, cardiac arrest patients; (4) reviews and case reports; and (5) meta-analysis, systematic reviews.

### Data collection and quality assessment

Two researchers independently extracted the following study design and patient characteristics: author name, type of surgery, study title, number and age of patients in both groups, surgical interventions, and correlation data between the two groups.

Two investigators independently assessed the quality of studies using the Cochrane Risk of Bias tool, which included six domains of bias: selection bias, performance bias, detection bias, attrition bias, reporting bias, and other bias (21). The investigators classified these studies as low, unclear, or high-risk. A study is defined as a low-bias risk only if the risk of bias is low in all six domains. If there were disputes about the reports of the included studies, discrepancies in the data extraction process were resolved by discussion with the staff of the scientific Research section.

### Outcomes

The primary outcome was postoperative delirium, and the second outcomes included the length of ICU stay, the incidence of kidney-related adverse outcomes, and the incidence of cardiac-related adverse outcomes.

### Statistical analysis

All data were meta-analyzed using Stata 16.0, and the random effects model was used to collect the data for pin-two meta-analysis. Egger's and Begg's tests were used to evaluate publication bias. If there is publication bias, the scissor-compensation method is used to investigate the publication bias. The Q (*P* < 0.1 indicates statistically significant heterogeneity) and I^2^ tests (I^2 ^> 50% indicates considerable heterogeneity) were used to evaluate the heterogeneity of the selected studies. I^2^ values of approximately 25%, 50%, and 75% were considered low, moderate, and severe heterogeneity, respectively (I^2^ > 50% was used as the threshold to indicate significant heterogeneity in individual studies). Data analysis of the collated data was performed using a fixed-effects model when I^2^ ≤ 50, and a randomized-effects model when I^2^ > 50. If I^2^> 50, meta-regression and subgroup analyses were performed to explore the source of heterogeneity. Descriptive analyses were performed if they could not be explained. *P* < 0.05 was considered statistically significant.

## Results

### Literature screening

**Figure 1:**
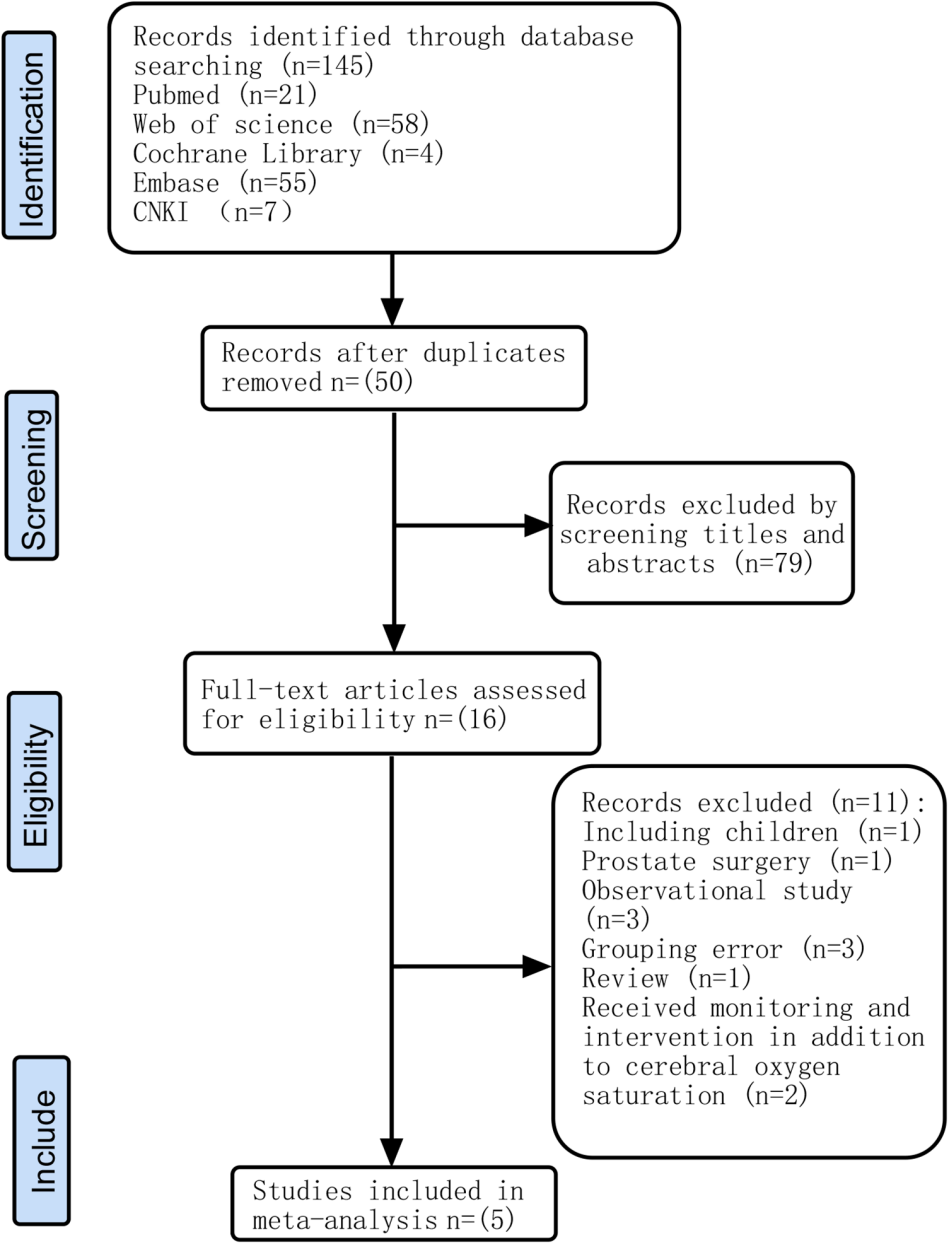
Flow chart of the study selection process.

A total of 145 relevant articles were retrieved according to the search terms, and 129 were excluded. After reviewing the relevance of the remaining 16 articles, we excluded 11 articles for the reasons shown in Figure 1. Five RCTs (22–26) involving 915 adult patients undergoing extracorporeal circulation surgery were included in this meta-analysis.

### Study patients and intervention characteristics

Of the 915 patients, 452 were assigned to the intervention group and 463 to the control group. In the five articles (22–26) we included, the definition criteria of cerebral oximetry desaturation in each study vary from below 75%–80% of baseline value or below 50%–60% of absolute value. The characteristics of the included studies are presented in Table 1.

**Table 1:** The characteristics of included studies.

Study	Sample size	Age (y)	Surgery	Intervention	CPB time	Outcome
Colak et al. 2015	94 VS. 96	61.9 ± 7.1 VS. 63.4 ± 8.8	CABG	rSO2 decreased below 80% of baseline value or below 50% of the absolute value	91 ± 31 VS 89 ± 32	POD
Lei et al. 2017	123 VS. 126	74.2 ± 6.5 VS. 72.9 ± 6.3	Valvular surgery or CABG	rScO2 decreased below 75% of baseline for 1 min or longer	109.1 ± 39.8 VS 115.8 ± 47.2	POD
Zavareh et al. 2019	111 VS. 112	62.02 ± 10.34 VS. 59.98 ± 11.19	Valvular surgery or CABG	the absolute threshold of rScO2 was less than 50%	56.95 ± 17.92 VS 59.85 ± 24.89	POD
Uysal et al. 2020	59 VS. 66	57.0 ± 11.0 VS. 58.0 ± 12.0	Cardiac surgery	rSO2 decreased below 60% of the absolute value than 60 consecutive seconds	132.8 ± 39.5 VS 138.4 ± 50.0	POD
Cheng et al. 2020	65 VS. 63	67 ± 8.2 VS. 67 ± 6.7	Valvular surgery or CABG	rSO2 decreased below 80% of baseline value for 1 min or longer	106 ± 38.7 VS 111.4 ± 33.4	POD

CABG, coronary artery bypass graft; POD, postoperative delirium; rSO2, regional cerebral oxygen saturations; CPB, cardiopulmonary bypass.

### Quality assessment

**Figure 2:**
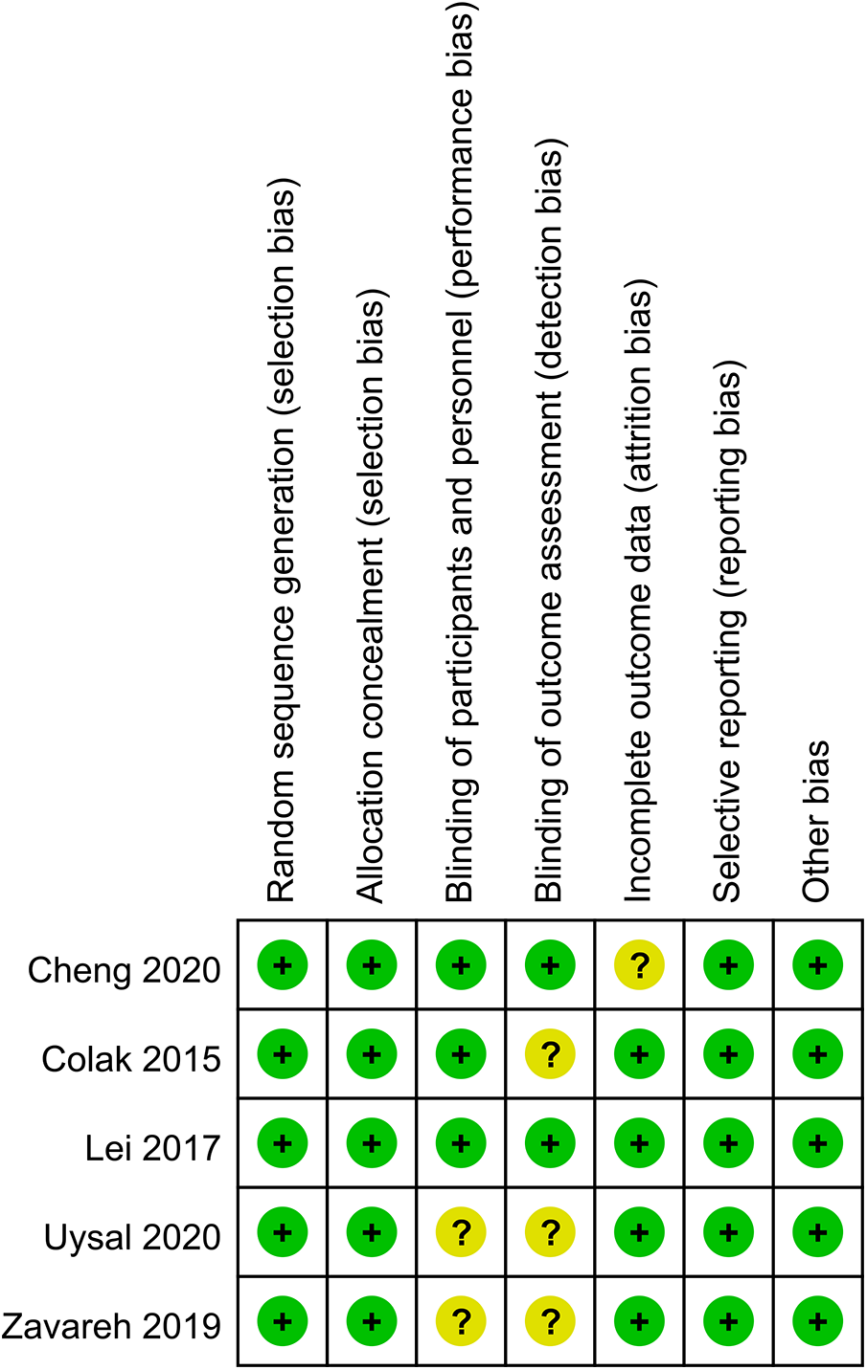
Risk of bias summary.

**Figure 3:**
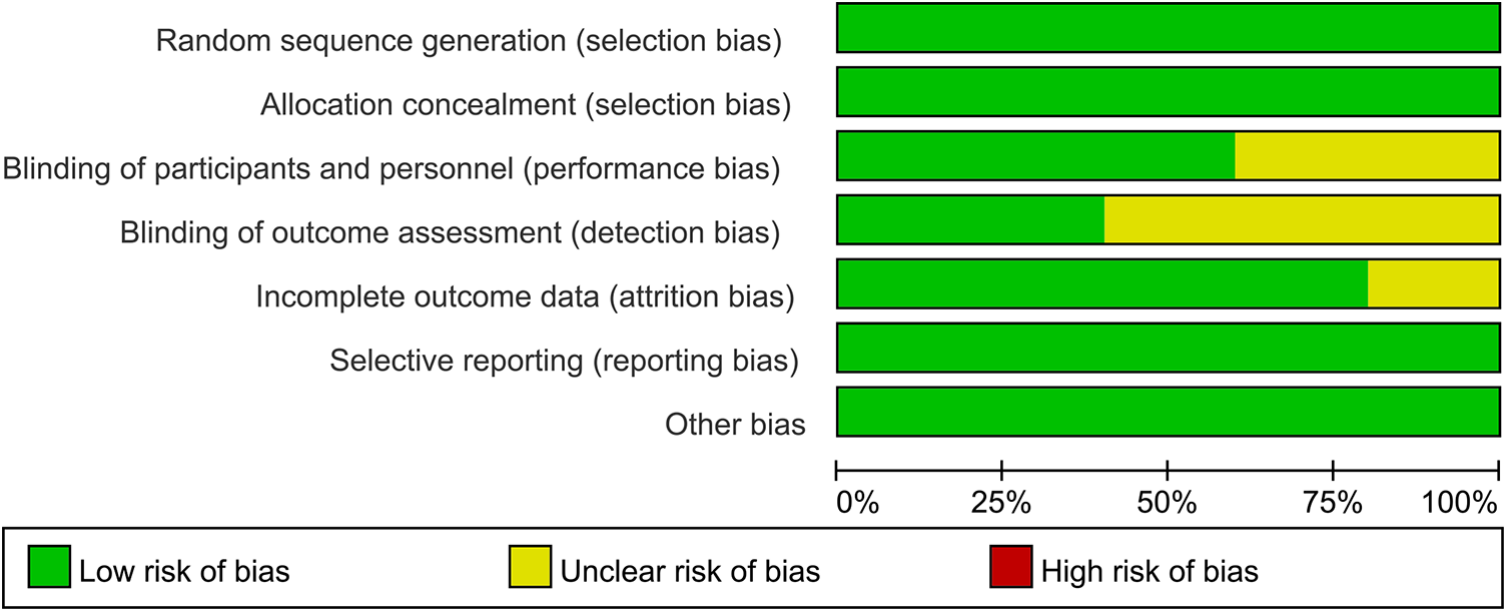
Risk of bias graph of each study.

In 2 out of 5 articles, the blinding of participants and personnel was unclear (25, 26). In 3 out of 5 articles, the blinding of outcome assessment was unclear (22, 25, 26). In 1 out of 5 articles, the incomplete outcome data were unclear (24). However, on the whole, there was only one low-risk article, which accounted for a relatively low proportion of the total number of articles. The quality evaluation of the included studies is shown in Figures 2, 3.

### Primary outcome

**Figure 4:**
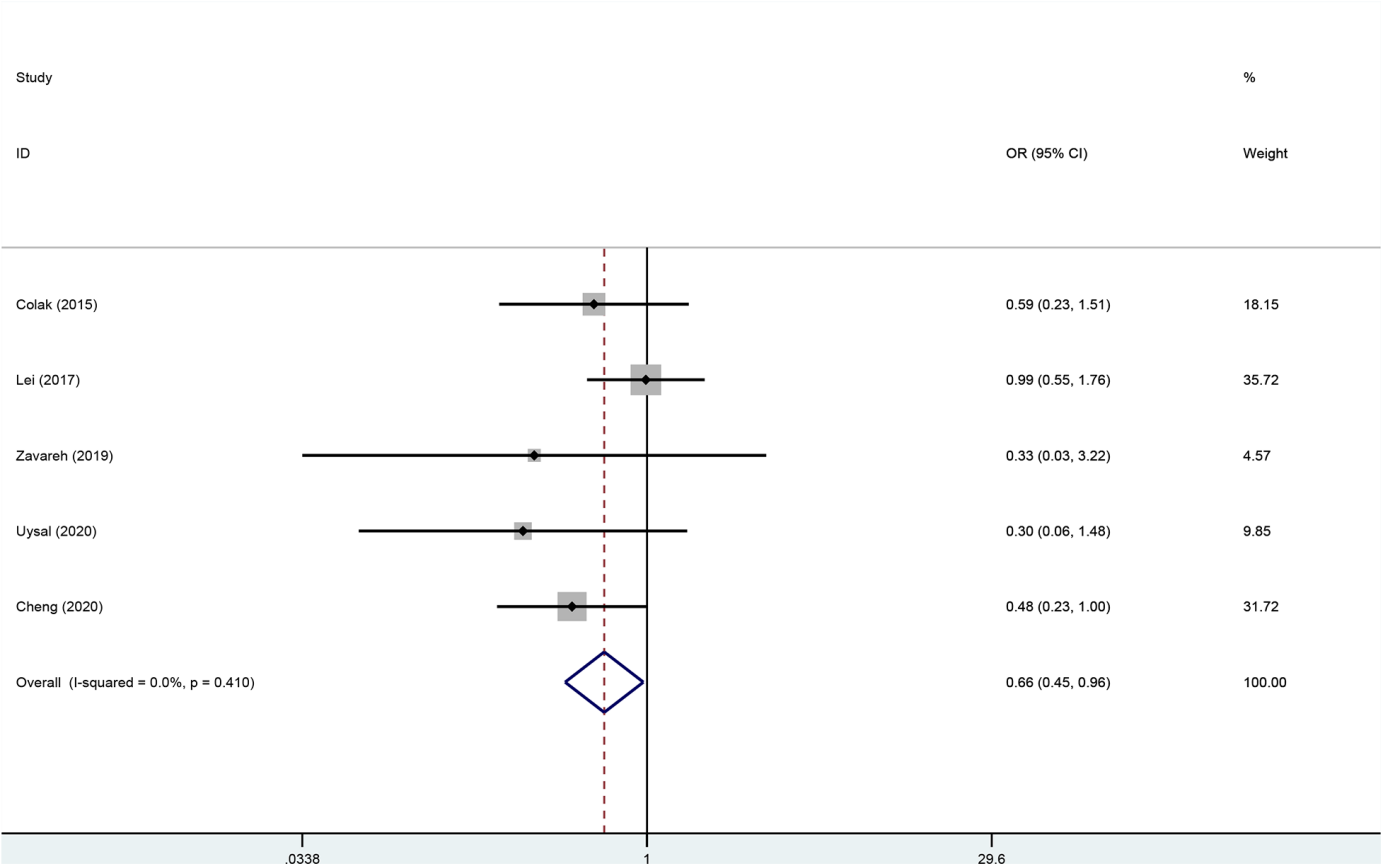
Forest plot of the effect of near-infrared spectroscopy on postoperative delirium in patients undergoing cardiac surgery with cardiopulmonary bypass.

Five studies (22–26) including 915 patients undergoing cardiopulmonary bypass reported the outcome of incidence of postoperative delirium between the rSO2 monitoring group (*n* = 452) and the routine care group (*n* = 463). The incidence of postoperative delirium in the intervention group was significantly lower than that in the control group (OR, 0.657; 95% CI, 0.447–0.965; *P* = 0.032; I^2^ = 0%) (Figure 4).

### Secondary outcomes

**Figure 5:**
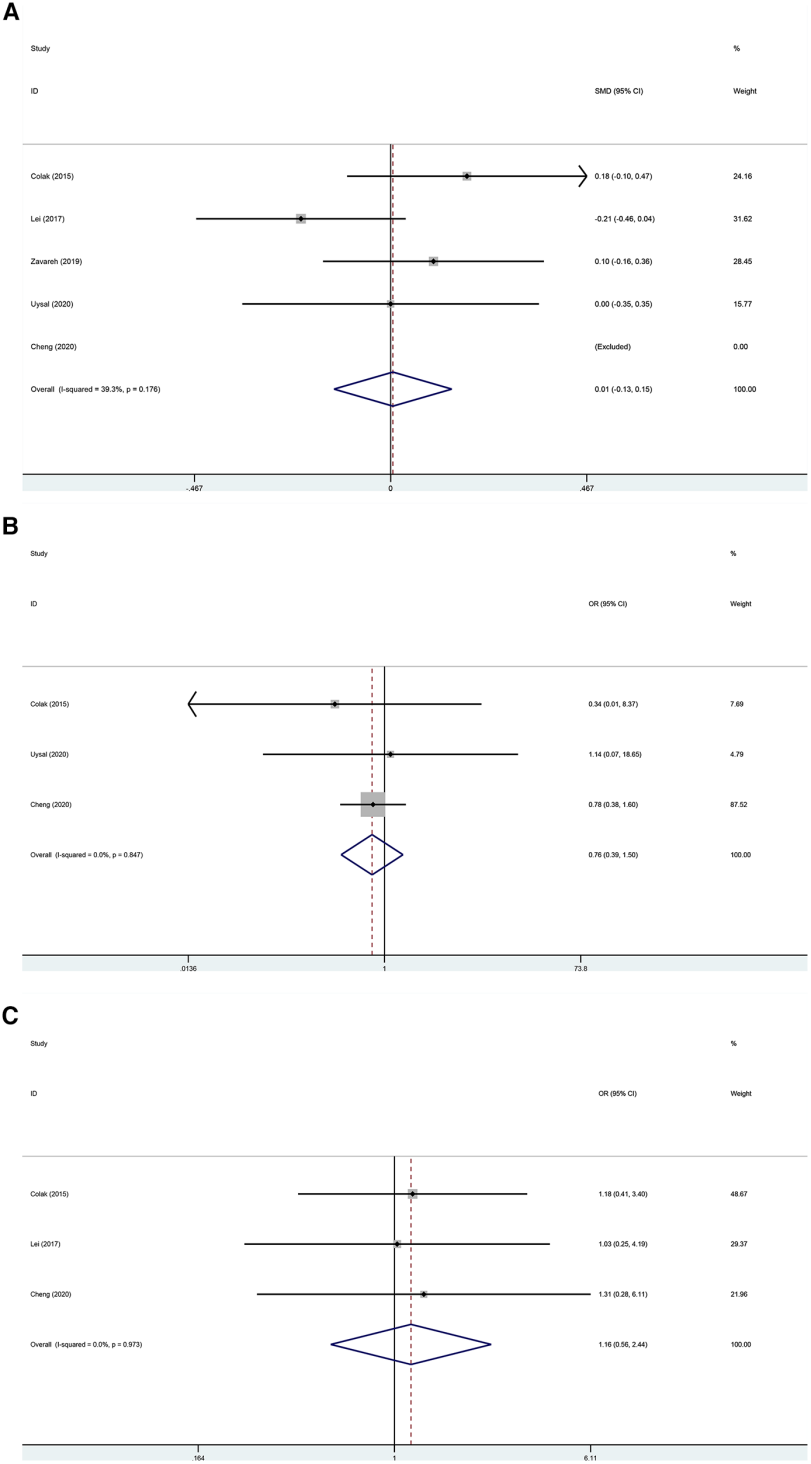
(**A)** Forest plot of the effect of near-infrared spectroscopy on ICU stay in patients undergoing cardiac surgery with cardiopulmonary bypass. (**B**) Forest plot of the effect of near-infrared spectroscopy on the incidence of kidney-related adverse outcomes in patients undergoing cardiac surgery with cardiopulmonary bypass. (**C**) Forest plot of the effect of near-infrared spectroscopy on cardiac-related adverse outcomes in patients undergoing cardiac surgery with cardiopulmonary bypass.

The postoperative ICU stay was examined in five trials (22–26) without a statistically significant difference between the two groups (Figure 5A; SMD, 0.005 days; 95% CI, −0.135 to 0.146; *P* = 0.940; I^2^ = 39.3%). The kidney-related adverse outcomes were examined in three trials (22, 24, 25) without a statistically significant difference between the two groups (Figure 5B; OR, 0.761; 95% CI, 0.386–1.500; *P* = 0.430; I^2^ = 0%). The cardiac-related adverse outcomes were examined in three trials (22–24) without a statistically significant difference between the two groups (Figure 5C; OR, 1.165; 95% CI, 0.556–2.442; *P* = 0.686; I^2^ = 0%).

### Subgroup analysis

**Figure 6:**
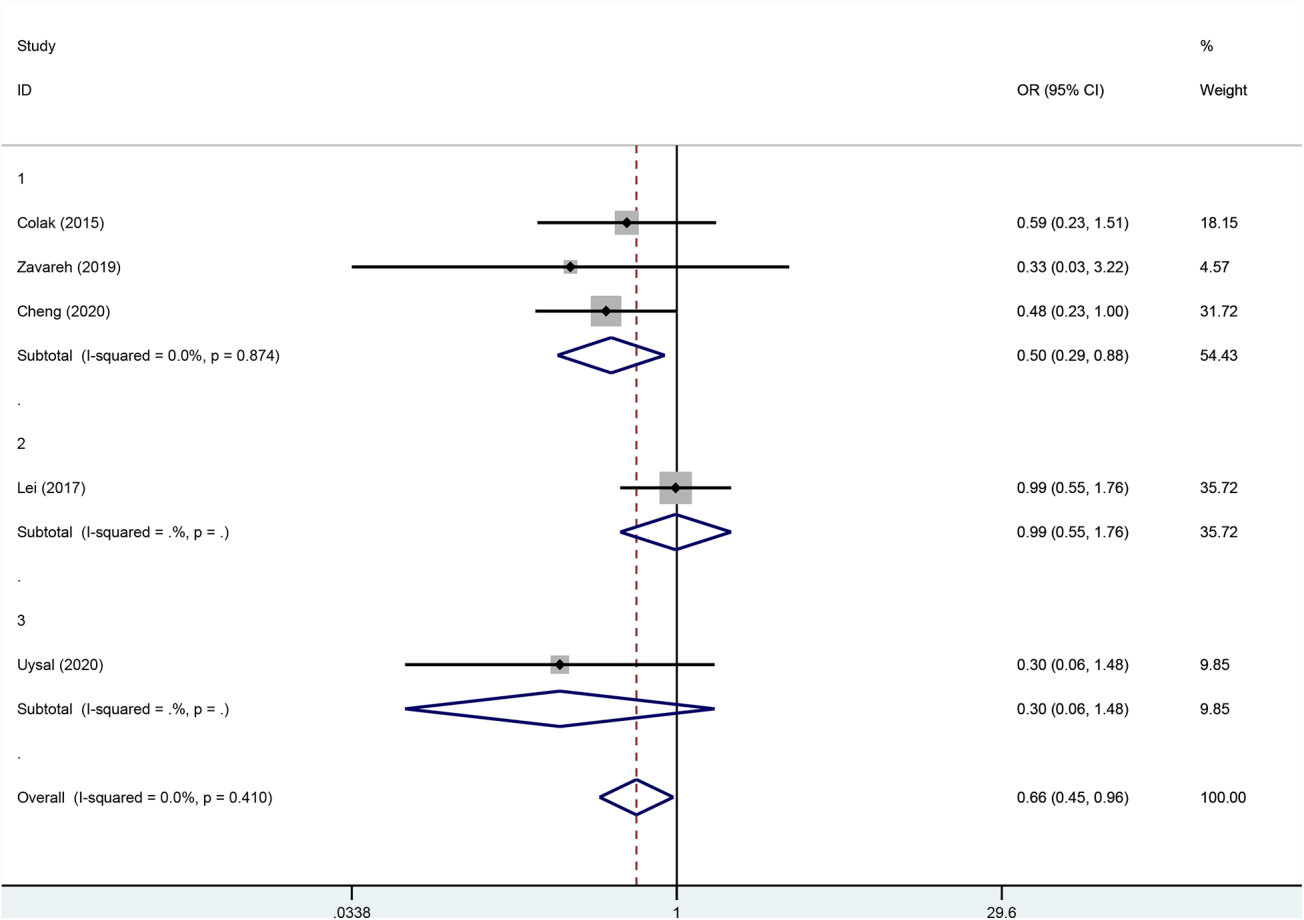
Subgroup analysis according to the different intervention premises.

Although there was no statistical heterogeneity in the primary outcome, subgroup analyses were performed to assess the robustness of the conclusions because of differences in the prerequisites for the clinical intervention. The result showed that when rSO2 fell below 80 or 75% of baseline values or below 50% or 60% of absolute values, the heterogeneity of primary outcomes did not change (Figure 6).

### Results of publication bias

According to the principle of publication bias, we counted articles whose outcome was postoperative delirium. Data analysis showed the incidence of postoperative delirium (Begg's *P* = 0.806 and Egger's *P* = 0.151), indicating that the article had no publication bias. The result of publication bias is shown in Table 2.

**Table 2:** Evaluation of publication bias and sensitivity analysis.

Index	OR (95%CI)	Z	*P*-value	I^2^(%)	I^2^'s P	Egger's P	Begg's P
POD	0.657[0.447, 0.965]	2.14	0.032	0	0.410	0.151	0.806

OR, odds ratio; 95% CI, 95% confidence interval; POD, postoperative delirium.

## Discussion

Delirium refers to a group of syndromes, also known as acute brain syndrome. It is manifested as consciousness disorder, disorderly behavior, and inability to concentrate. Usually, the onset of the disease is urgent, and the disease fluctuations are obvious. The syndrome is common in elderly patients. The patient's cognitive function is decreased and the sensory perception is abnormal. Delirium is not a disease, but a clinical syndrome caused by a variety of causes (6). Cerebral oxygen saturation is considered to be a major factor in postoperative delirium in surgical patients. In cardiac surgery with cardiopulmonary bypass, the formation of inflammatory responses, hypoperfusion, and micro embolism associated with cardiopulmonary bypass is more likely to lead to the decrease of cerebral oxygen saturation in patients during surgery (8), and this relationship also affects the increased incidence of postoperative delirium in patients.

The effect of NIRS monitoring cerebral oxygen saturation on POD in patients undergoing cardiac surgery with CPB is uncertain. This is the first meta-analysis of the effect of NIRS monitoring on POD in patients undergoing cardiac surgery with CPB, which provides evidence for the use of NIRS in patients undergoing cardiac surgery with CPB. In this meta-analysis, we selected cardiac surgery under limited conditions of CPB. A total of 915 patients with postoperative delirium were evaluated, including 452 in the intervention group and 463 in the control group. This meta-analysis showed that intraoperative CPB guided by the monitoring of cerebral oxygen saturation could help reduce postoperative delirium in patients. In addition, the data analysis of the five studies included in our study showed that the heterogeneity was not high, so we did not conduct a subgroup analysis. When we collected and collated the data, The definition criteria of cerebral oximetry desaturation in each study varied from below 75%–80% of baseline value or below 50%–60% of absolute value. Studies have also found that long-term low rSO2 leads to an increase in the incidence of POD (27). Therefore, intraoperative rSO2 monitoring can reduce the incidence of POD and improve the postoperative quality of life.

It has been suggested that mechanisms supporting monitoring and intervention of changes in cerebral oxygen saturation during cardiac surgery to prevent postoperative delirium may include, clinically, a brain injury that may involve cerebral hypoperfusion (28–31). In the brain tissue, drastic changes in blood flow lead to changes in cerebral oxygen saturation, and the cerebral autoregulation (CA) response can prevent brain injury caused by drastic changes in cerebral perfusion (32). According to previous studies, CA disorders occur in 20% of patients undergoing CPB (33). During surgery, heart flipping can temporarily affect cardiac output, thereby causing changes in cerebral perfusion (34). In addition, during CPB, if the fresh gas delivered to the membrane oxygenator in the circuit does not change at the beginning of mechanical ventilation, hypocapnia can easily lead to cerebral vascular contraction, resulting in drastic changes in cerebral perfusion (35). Studies have also shown that low temperature during CPB can lead to increased cerebral blood flow and exceed the normal demand of brain tissue metabolism, thereby destroying the CA (36). Similarly, rewarming can lead to CA disorder (37, 38). Burst inhibition of sevoflurane, an inhaled anesthetic, can lead to reactive vasodilation, affecting the CA during CPB (39). Other studies have found that diffuse microemboli are more subtly associated with POD and that CPB surgery is more likely to result in more microemboli than off-pump surgery (8, 40). The presence of these clinical factors may lead to the possibility of POD. Brain ischemia leads to rapid depletion of energy stores, causing cell depolarization and Ca^2+^ influx, leading to excitotoxic cell death and thus resulting in hypoxic-ischemic neuronal damage (41, 42). NIRS can not only reflect the baseline level of cerebral oxygen, but also monitor the fluctuation of cerebral oxygen saturation during operation, which can reflect cerebral perfusion. Anesthesiologists can pretreat according to cerebral oxygen saturation to correct possible hypotension and hypoperfusion, prevent cerebral ischemia and hypoxia, and therefore may prevent POD. Under the monitoring of NIRS, CPB can intervene with the POD caused by oxidative stress through changes in cerebral oxygen saturation, increasing cerebral perfusion, using vasoactive drugs, adjusting the depth of anesthesia, rehydration, and other measures.

However, there are limitations to this meta-analysis. The limitations of monitoring instruments NIRS, especially in terms of monitoring techniques and algorithms that vary from model to model, also add to the heterogeneity and instability of results. Other factors such as skull thickness, extracranial tissue density, and other differences between individuals may also lead to different monitoring results (43). In addition, areas with cerebral hypoperfusion or delayed clearance of microemboli during CPB may be far from the location of the NIRS monitoring area, resulting in false negative results (44). At the same time, it has been reported in the literature that NIRS monitoring results in deceased patients showed normal oxygen saturation in the brain, leading some researchers to question the accuracy of NIRS (45, 46). Therefore, some researchers no longer use absolute values to determine cerebral hypoperfusion, but rather relative measures such as automatic brain regulation (47). There were different scales to assess the incidence of POD in our included studies, such as CAM/CAM-ICU, Criteria and Diagnostic Manual of the Diagnostic and Statistical Manual of Mental Disorders, MMSE, and other scales that had different specificity and sensitivity. The definitions for cerebral desaturation are different among the included trials.

Due to the inconsistency of these prerequisites, insufficient sample size, and the limitation of near-infrared spectroscopy, the comparability of results is greatly affected, and the study of cerebral oxygen saturation has a long way to go. Only publications in English were included, which could lead to potential publication bias. However, the assessment of publication bias was not significant. Finally, all five included studies were randomized controlled trials with small sample sizes. To clarify intraoperative interventions and optimize the neuroprotective effect of cerebral oxygenation in patients undergoing cardiac surgery, further randomized controlled trials with large sample sizes are needed.

## Data Availability

The original contributions presented in the study are included in the article/Supplementary Material, further inquiries can be directed to the corresponding author.
